# Neural basis of forward flight control and landing in honeybees

**DOI:** 10.1038/s41598-017-14954-0

**Published:** 2017-11-06

**Authors:** M. R. Ibbotson, Y.-S. Hung, H. Meffin, N. Boeddeker, M. V. Srinivasan

**Affiliations:** 1 National Vision Research Institute, Australian College of Optometry, Carlton, Victoria Australia; 2 Department of Optometry and Vision Sciences, University of Melbourne, Parkville, Victoria Australia; 3 National Institute of Child Health and Human Development (NICHD), National Institutes of Health (NIH), 9000 Rockville Pike, Bldg 35 A, Bethesda, MD USA; 4 Department of Cognitive Neuroscience, Bielefeld University, 33615 Bielefeld, Germany; 5 Queensland Brain Institute, University of Queensland, St Lucia, QLD 4072 Australia

## Abstract

The impressive repertoire of honeybee visually guided behaviors, and their ability to learn has made them an important tool for elucidating the visual basis of behavior. Like other insects, bees perform optomotor course correction to optic flow, a response that is dependent on the spatial structure of the visual environment. However, bees can also distinguish the speed of image motion during forward flight and landing, as well as estimate flight distances (odometry), irrespective of the visual scene. The neural pathways underlying these abilities are unknown. Here we report on a cluster of descending neurons (DNIIIs) that are shown to have the directional tuning properties necessary for detecting image motion during forward flight and landing on vertical surfaces. They have stable firing rates during prolonged periods of stimulation and respond to a wide range of image speeds, making them suitable to detect image flow during flight behaviors. While their responses are not strictly speed tuned, the shape and amplitudes of their speed tuning functions are resistant to large changes in spatial frequency. These cells are prime candidates not only for the control of flight speed and landing, but also the basis of a neural ‘front end’ of the honeybee’s visual odometer.

## Introduction

Forward movement through the world generates distinct optic flow fields on the retinas: expansion from a central point in the frontal visual field and back-to-front motion over the lateral (or peripheral) parts of the visual field^1^. These flow fields are experienced by insects as they fly through their visual environments and when they land. Honeybees use visual cues to control flight speed as well as the attitude they fly^2^. In open flight they maintain a constant front-to-back image speed over their retinas of 215–320°/s^2–4^, and they streamline the pitch of their abdomen at these image speeds^5^. Riley *et al*.^6^ similarly found that cruising bumblebees maintain a constant rate of image flow past their eyes of approximately 200°/s. Honeybees also use similar strategies when landing on planes of different orientations. When landing on vertical surfaces, they adjust their forward flight speed to maintain a constant rate of expansion of about 220°/s in the fronto-lateral visual field^7^ (i.e. in a viewing direction 45° away from ‘straight ahead’). Similarly, when landing on horizontal surfaces they adjust their forward speed as they descend, such that the rate of motion of the image of the surface in the ventral visual field is held constant^8^. These strategies ensure that as the bee approaches the surface its flight speed automatically decreases, reaching near-zero at the point of contact. Honeybees search for food over many kilometers, and once a food source is identified they fly home along the shortest route and signal the direction and distance of the food source to their hive mates using a waggle dance^9,10^. The distance to the food source is signaled by the duration of the dance and it is thought that the bee’s ‘neural odometer’ works by integrating, over time, the image speed experienced during the flight to obtain an estimate of the distance traveled^11,12^. The range of behavioral experiments described above has shown that even large changes in the spatial frequency content of the visual environment have little influence on these speed-related behaviors^13^. This is different to other visuo-motor responses in bees, such as the optomotor turning response, where the rotational torque is optimal at different speeds depending on the spatial frequency used^14^.

Our goal was to understand more about the underlying neural pathways involved in forward flight control and landing in bees. We recorded from descending neurons (DNs) that carry visual motion information from the central brain to the thoracic motor centers. Direction selective DNs have been identified in many insect species (e.g. bees^15–17^, flies^18–21^, lepidoptera^22^, locusts^23–25^, praying mantis^26^, and dragonflies^27,28^). As all these DNs integrate directional information from large patches of the visual field, they are excellent candidates for an investigation of the neural basis of forward flight control. Via intracellular staining of DN axons in the bee ventral nerve cord, Goodman *et al*.^29^ observed six types of DNs each with distinct dendritic shapes and cell body locations in the brain (categorized as groups denoted by Roman numerals: DNI to DNVI). Later, it was shown that neurons in all of these groups had terminal axon collaterals in the pro- and mesometathoracic ganglia^15,30–33^. Measurement of the directional tuning properties of these neurons, along with another neuron (DNVII) that did not fit into the six original categories^31^, revealed that they all respond strongly to specific optic flow fields. The conclusion from this work is that most of the cell groups are involved in signaling information relevant for optomotor body stabilization during unintentional body rotations (i.e. roll, yaw or pitch).

One of the original cell groups, labeled DNIII^29^, has received less attention than the others. The brain and thoracic anatomy, along with the directional tuning properties of a single DNIII neuron were reported^31^. It responded best to horizontal image motion from front-to-back over the lateral portions of both eyes. Bidwell and Goodman^34^ used a frontally located split-screen stimulus to show that two types of DNIII neuron responded to frontal expansion, but that study did not explore their speed tuning properties, nor did they present any anatomy to verify their identification. Here we present the anatomical characteristics of the DNIII neurons to better understand their connectivity in the brain and thoracic ganglia. We present data collected from anatomically identified DNIII neurons to assess their directional and speed tuning characteristics. The cells are not strictly speed tuned, as changes in spatial frequency influence the response amplitude, however the speed and directional tuning of the cells matches those required for forward flight control, including landing.

## Methods and Materials

### Experimental animals

Experiments used worker honeybees, *Apis mellifera*, collected from hive entrances or wild-caught in local parks. Bees were anaesthetized by cooling in a refrigerator at 5 °C for 20 minutes before each experiment. General experimental procedures were identical to those in our previous study^16^. Bees were placed horizontally (dorsal side up) in a metal holder. The head and thorax were secured with a 3:1 mixture of beeswax and violin resin. A chlorinated silver wire was inserted into the thorax as an indifferent electrode. The ventral nerve cord was exposed from the dorsal side of the neck, between the suboesophageal and prothoracic ganglia. After preparing the bee it was left for 15 minutes to recover to room temperature (22–24 °C).

Microelectrodes were pulled using a P-97 Flaming/Brown puller (Sutter Instruments) from borosilicate glass capillaries (GC100TF-10, Harvard Apparatus LTD). Electrode tips were filled with 10% Lucifer Yellow CH (lithium salt; Invitrogen) in 1% LiCl solution and backfilled with 1 M LiCl solution. Electrode impedance was approximately 100 MΩ.

Electrodes were inserted vertically into the exposed ventral nerve cord using a mechanical micromanipulator. Monitoring the measured electrode potential on an oscilloscope assessed successful axon penetration. When a cell’s resting potential was detected, wide-field visual stimuli were presented to stimulate the eyes and confirm that the cell was visually responsive.

### Visual Stimulation

**Figure 1:**
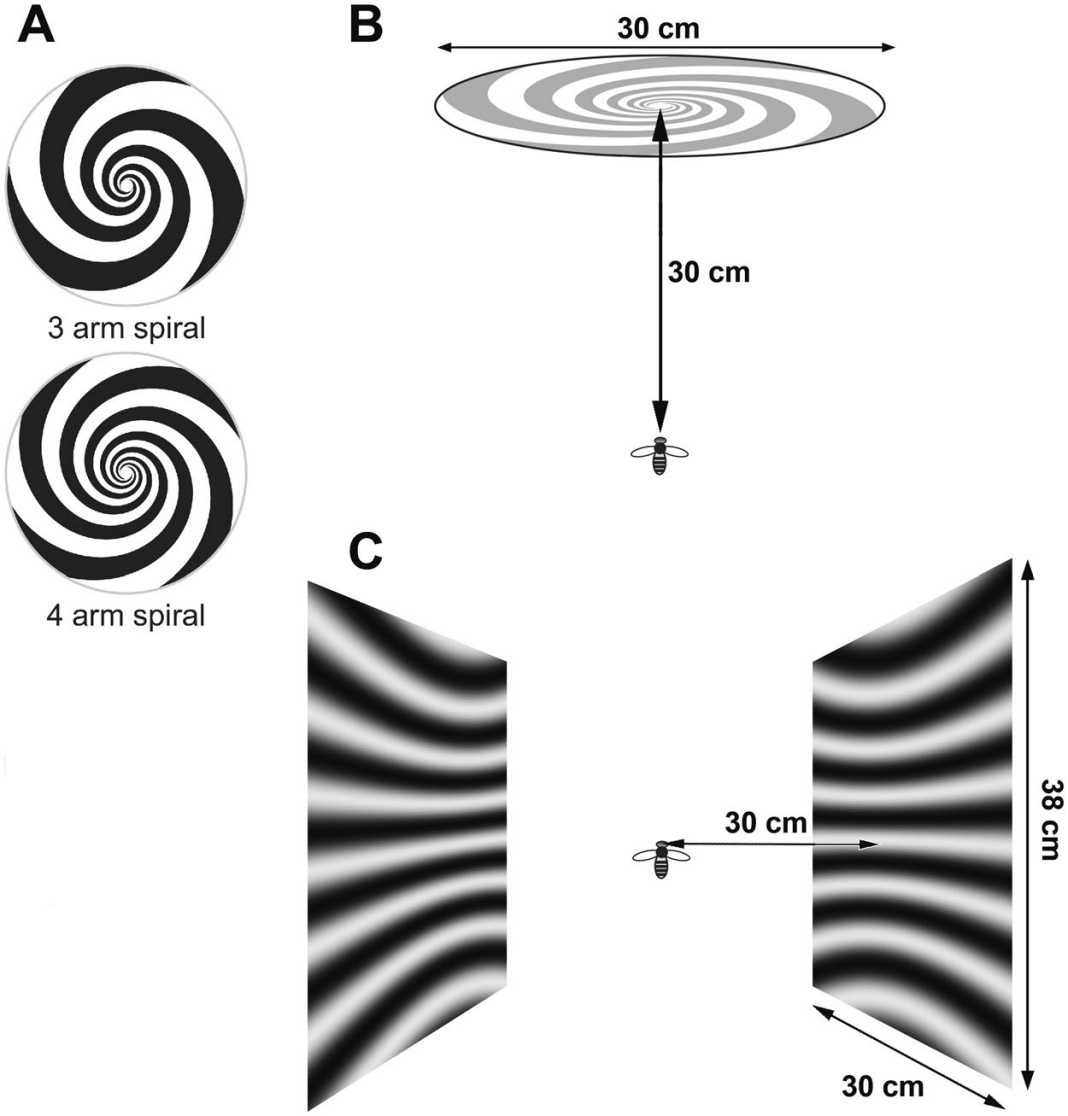
Stimulus configurations. (**A**) 3-arm and 4-arm spirals used for frontal stimulation. (**B**) Positioning of the bee relative to the frontal spiral stimulus. (**C**) Illustration of the lateral stimulus screens.

Neurons were tested for visual responses quantitatively by presenting stimuli on CRT display monitors, driven by VSG Series 2/5 stimulus generators (Cambridge Research Systems, Cambridge, UK). The monitors were gamma-corrected and ran at 198 Hz (Clinton Monoray, 57 cd/m^2^ mean luminance, 600 × 400 pixels) at a viewing distance of 30 cm. In one set of experiments a single monitor was placed directly in front of the bee (Fig. 1A,B). In a second set of experiments we used two monitors, placed on each side of the bee’s head (Fig. 1C). Stimuli were either high contrast (0.6) sine-wave gratings that could be moved back and forth at any orientation on the screen (Fig. 1C) or spiral patterns that could be rotated clockwise or anticlockwise to generate expansion or contraction of the frontal image (Fig. 1A,B). A range of spatial and temporal frequencies were used for the linear sine wave gratings, as follows: temporal frequencies: 2, 3, 4, 6, 8, 12, 16, 24, 32, 48 Hz; spatial frequencies: 0.071, 0.047, 0.035 and 0.028. Spatiotemporal tuning functions show white lines of constant speed (50°/s, 100°/s, 200°/s, 400°/s and 800°/s).

Spiral patterns were only used on the frontal screen. We used exponential spirals, in which the radius increases exponentially with the angle. The reason for the choice of an exponential spiral (as opposed to an Archimedian spiral, for example) is that a rotating exponential spiral generates exactly the same pattern of optic flow that a visual system would experience when it approaches a stationary vertical surface^7^. Spirals with 3 or 4 arms were used (Fig. 1A), rotating at up to 5 rps, with an exponential pitch of 0.3. The geometry of the spirals, and the pattern of image motion that they generate is described in the *Supplementary Information*.

### Data analysis

The electrode potential was amplified (Getting Instruments, Model 5 A), displayed on an oscilloscope and recorded digitally at a sampling rate of 5 kHz, on a PC using a 14-bit analogue-to-digital converter (Measurement Computing, PCI-DAS1001). The recorded data was analyzed offline using MATLAB. A spike was judged to have occurred if the difference between consecutive measurements of the membrane potential (dV) exceeded a threshold. This threshold was set at three times the standard deviation of dV over the entire recording. Spikes were determined to occur at the zero crossing of dV that occurs immediately following the point at which dV exceeds the threshold. The spike arrival times were then used to calculate perisitimulus time histograms (PSTHs). The bin widths of the PSTHs are given in the text and figure legends. For some data, we calculated spike density functions (SDFs) with 1 kHz resolution to better reveal the detailed response waveforms of the neurons. The SDFs were created by convolution of a Gaussian kernel of unit area and $\sigma =8\,$ms with a train of Dirac delta functions; one delta function corresponding to the arrival time of each spike. Mean SDFs were then calculated by trial averaging responses to individual stimulus presentations. The spontaneous firing rate of each neuron was estimated by averaging spike rate over periods of 1 s immediately prior to each stimulus presentation during which the stimulus monitor displayed a mean gray screen.

### Anatomical analysis

After intracellular recording, cells were filled with Lucifer Yellow CH (lithium salt, Invitrogen^TM^) by iontophoretic injection of 5–10 nA (negative injection) for at least 15 minutes. To understand the morphology of motion-sensitive descending neurons in the central brain and thoracic ganglions, we performed fluorescent mass staining of the bee ventral nerve cord. In those experiments, a glass pipette filled with Texas Red (Invitrogen^TM^) was inserted dorsally into the ventral nerve cord for at least 2 hours to allow dye uptake and diffusion through the neurons. In both cases, following a general histological protocol^16^, the honeybee brain, pro- and mesometa-thoracic ganglia were optically sectioned using a confocal microscope (Fluoview 1200, Olympus), with a 10x objective lens (NA = 0.4; UPLSAPO, Olympus). Images were collected with resolution of 0.8 μm per pixel. The image series were processed in ImageJ to produce 2D projections of the samples at different angles. In the mass staining experiments, the images of serial scanning (cross sections) were also projected every 5–10 μm to show neural structures at different depths. Compared with the projections of the filled single cell images, the axons and dendrites of the neurons of interest were manually traced out in the stacks of the projected images in Photoshop.

## Results

### Anatomy

**Figure 2:**
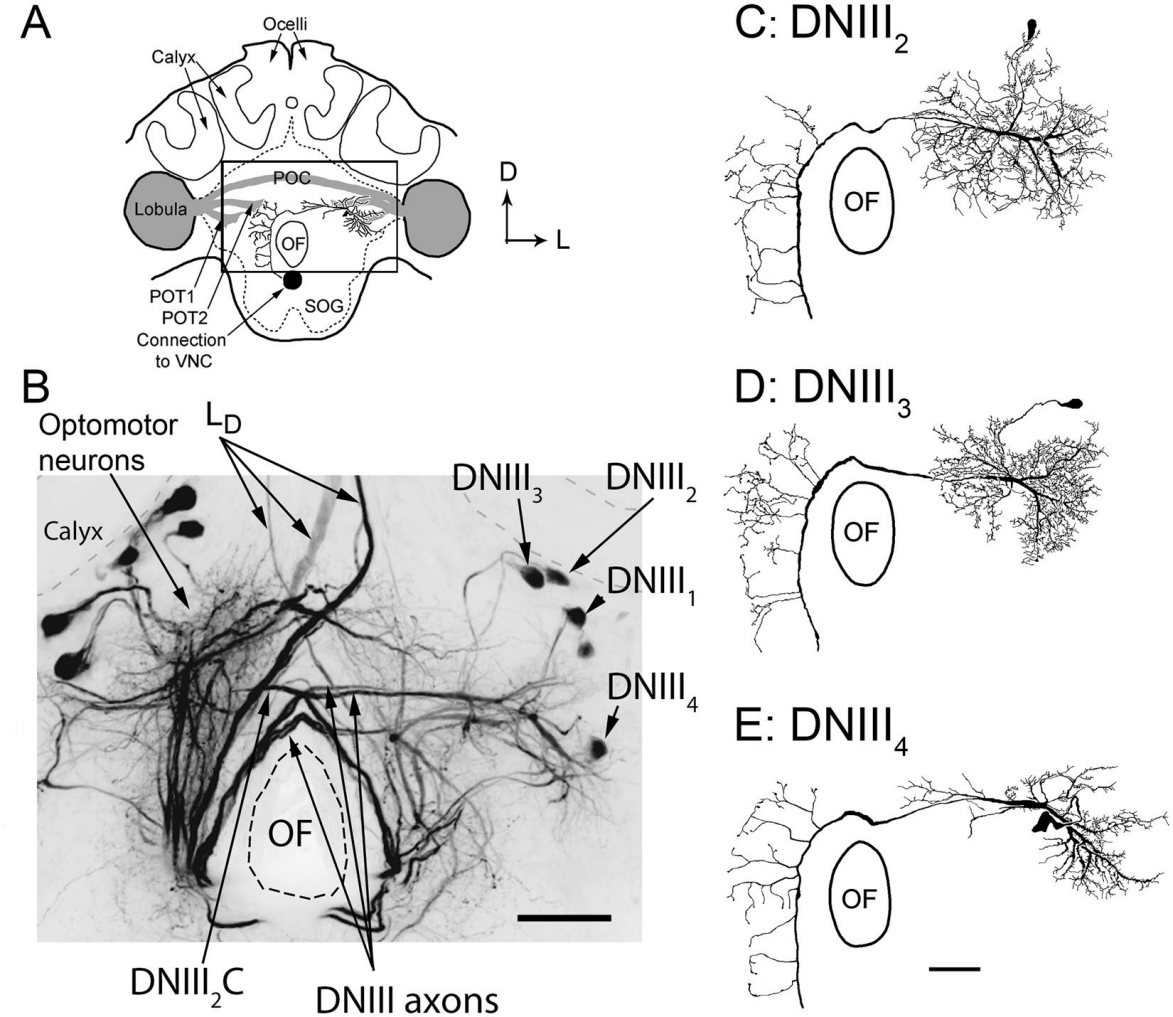
Brain anatomy. (**A**) A schematic diagram of the bee brain as viewed from the posterior perspective. The posterior optic tracts (POT1 and POT2) and the posterior optic commissure (POC) are shown in grey. The box shows the location of the image shown in B. (**B**) Photograph of a mass-fill obtained after placing dye in the ventral nerve cord. The optomotor neurons and the DNIII neurons are labeled. The axons of all four DNIII neurons in the left hemisphere of the brain were filled (labeled DNIII_1–4_), only one DNIII_2_ neuron was filled in the other side (labeled DNIII_2_C, with C = contralateral). (CDE) Drawings of high quality fills showing the anatomies of DNIII_2_, DNIII_3_ and DNIII_4_. A high quality fill was not obtained from DNIII_1_. Scale bars = 100 µm. OF = oesophageal foramen.

In bees, the anterior-posterior neuroaxis is tilted upwards by 90° in the head capsule^35^. Thus anterior regions of the brain, using neuroaxis terminology, become dorsal and posterior regions ventral, with respect to the body axis. Here, anatomical characteristics will be described with reference to the body axis. This makes descriptions relative to previous studies on DNs easier^15,16,31^. When dye is injected into the ventral nerve cord of the bee at a location just anterior to the prothoracic ganglion, a large number of DNs with their dendrites and cell bodies in the central brain are filled (Fig. 2A,B). In Fig. 2B, many DNs appear to the left of the oesophageal foramen (OF) and are labeled ‘optomotor neurons’. This general label includes cells from the DNII, DNIV and DNVI categories^29^ and the optomotor label derives from the fact that recordings from these neurons have revealed an ability to detect rotational image flow, as occurs during head roll, yaw and pitch^15,30,32^. The optomotor neurons have their dendrites and cell bodies in the most posterior 110 µm of the brain. Another prominent type of filled DNs are the ocellar L_D_ neurons, which have large axons that descend from the ocellar retinas (Fig. 2B; for details, see Hung and Ibbotson^16^).

In the present work, we will describe the anatomies and visual response properties of a specific class of DNs referred to as the DNIII group, which have their axons and dendrites in more anterior strata of the brain, approximately 110 to 140 µm below the posterior surface of the brain (Fig. 2B–E). Our mass-fill preparations have revealed that four DNIII neurons exist in each side of the brain. In Fig. 2B we filled the axons of all four DNIII neurons in the left side of the brain (labeled DNIII_1–4_) and just one DNIII neuron in the other side of the brain (labeled DNIII_2_C, with C = contralateral, note: its cell body is not visible). The cell bodies of the four neurons with axons in the left connective are labeled in the right hand side of the brain (DNIII_1_, DNIII_2_, DNIII_3_, DNIII_4_). We present drawings of the DNIII_2_, DNIII_3_ and DNIII_4_ neurons, as derived from single-cell fills (Fig. 2C,D,E). Good brain fills from 9, 5 and 8 cells were obtained from DNIII_2_, DNIII_3_ and DNIII_4_, respectively, and many more partial fills. We do not have a high quality fill of DNIII_1_, so no single drawing is presented. Partial fills of lower quality were obtained from this neuron but due to limitations in identifying it, we do not present physiological data from it.

All of the neurons in the DNIII group have dense dendritic fields situated in the lateral protocerebrum close to the exit of the lobula on the same side of the brain as the cell bodies (ipsilateral) (Fig. 2A–E). The dendrites are located close to the exit points from the anterior compartment of the lobula complex (often referred to as the ventral lobula using neuroaxis terminology). The DNIII axons pass over the dorsal portion of the OF. Once in the contralateral brain the axons send out many thin blebbed branches that invade the protocerebrum and perioesophageal neuropile (Fig. 2B–E). The DNIII_4_ neuron is quite unique because its cell body is located immediately posterior to its dendrites, such that when viewed from the posterior side of the brain the dendrites and cell body overlap (Fig. 2B,E). The DNIII_1_, DNIII_2_ and DNIII_3_ neurons have their cell bodies more dorsal, close to the calyces^29^ (Fig. 2B,C,D).

**Figure 3:**
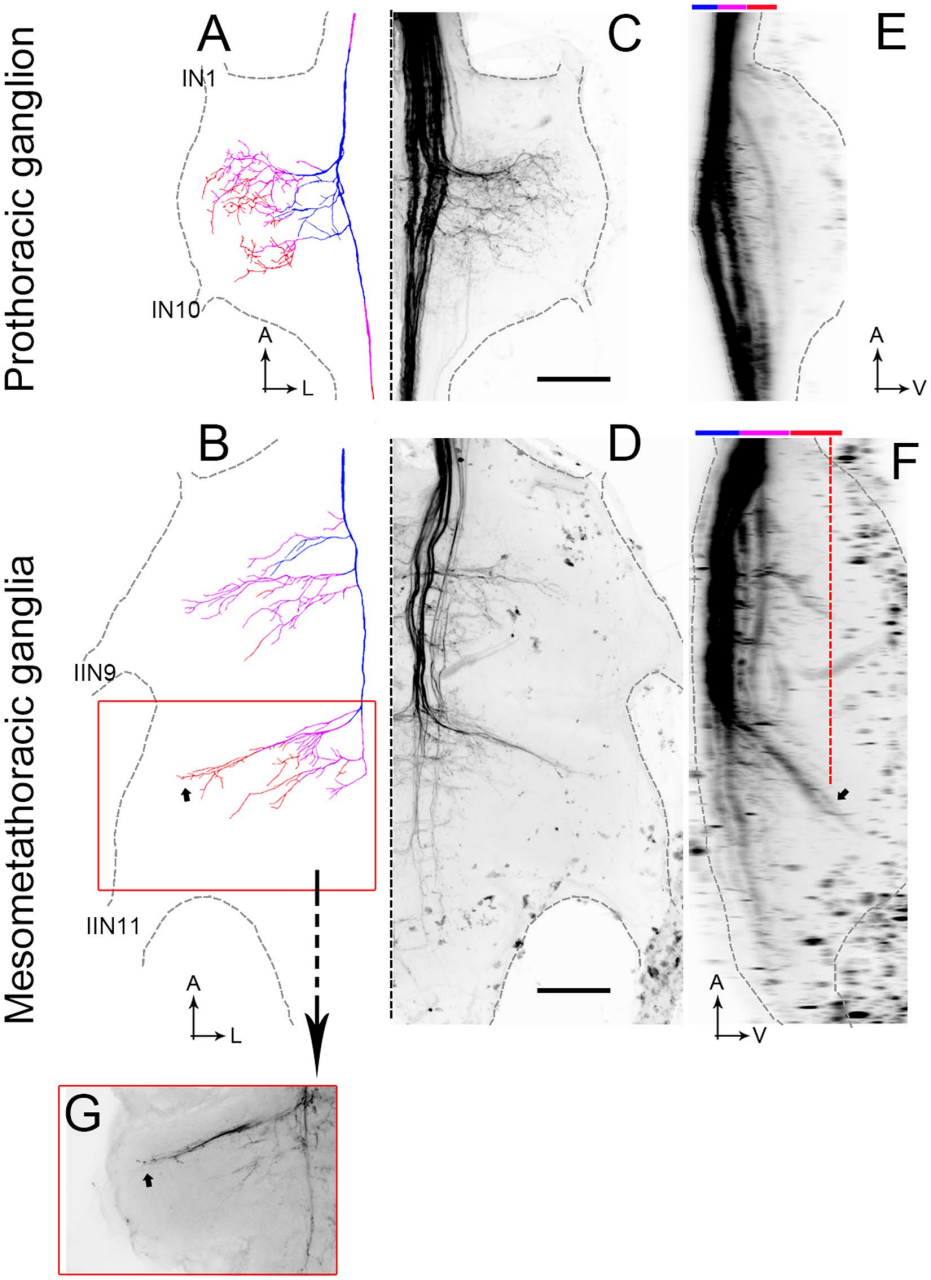
Thoracic anatomy. (**A**,**B**) Drawing of DNIII_4_ in the thoracic ganglia. Blue shows dorsal axon collaterals and orange shows ventral collaterals. (**C**,**D**) Mass-fills showing the DNIIIs and other descending neurons in the thoracic ganglia (reflection of drawings in (**A** and **B**). (**E**,**F**) Side views of the mass fills shown in (**C**) and (**D**). The upper color bars show the depths as represented in A and B. The small arrow in F shows the locations of the terminals indicated by the same small arrow in B. The dashed orange line in F shows the depth plane of the photograph taken in (**G**). (**G**) Photograph of the terminals highlighted by the small arrows in B and F. The orange box surrounding (**G**) is shown as an orange box in B. Scale bars = 100 µm. A = anterior, L = lateral. IN1 and IN10 are prominent nerves in the prothoracic ganglion. IIN9 and IIN11 are the largest nerves exiting the mesometathoracic ganglia.

It proved very difficult to get dye to flow into the thoracic ganglia. We have observed the four axons running together in the medial dorsal tract (MDT, as defined by Rehder^36^ 1988) as far as the most posterior end of the prothoracic ganglion. We have also observed the axons of some DNIII neurons running together in the MDT in the meso-metathoracic ganglion, however we could not identify them individually based on brain anatomy. We are therefore unable to describe the full thoracic anatomy of the whole cell group. However, DNIII_4_ was filled into the thoracic ganglia and simultaneously identified in the brain (Fig. 3A,B). The neuron is color coded to reveal the depths of the various axon segments. The axon descends into the thoracic ganglia through the medial dorsal tract (MDT). In the example shown, other DNIII neurons travel with it in the MDT, but it is not clear which specific cell types. The axon of DNIII_4_ gives off numerous blebbed branches into the axonal side of the ganglia (contralateral to the cell body) (Fig. 3A–D). The branches invade the dorsal and ventral regions of the ganglia, the ventral projections being revealed in the thoracic side views (Fig. 3E,F). The most noteworthy feature of the descending elements is that in the meso- and metathoracic ganglia (Fig. 3F) the branches reach ventrally towards the motor neuropiles for the middle and hind legs^37,38^. This characteristic is very different from previously described direction-selective bee DNs, which confine their axonal branches to the most dorsal quarter of the ganglia^15,30^.

### Directional tuning

**Figure 4:**
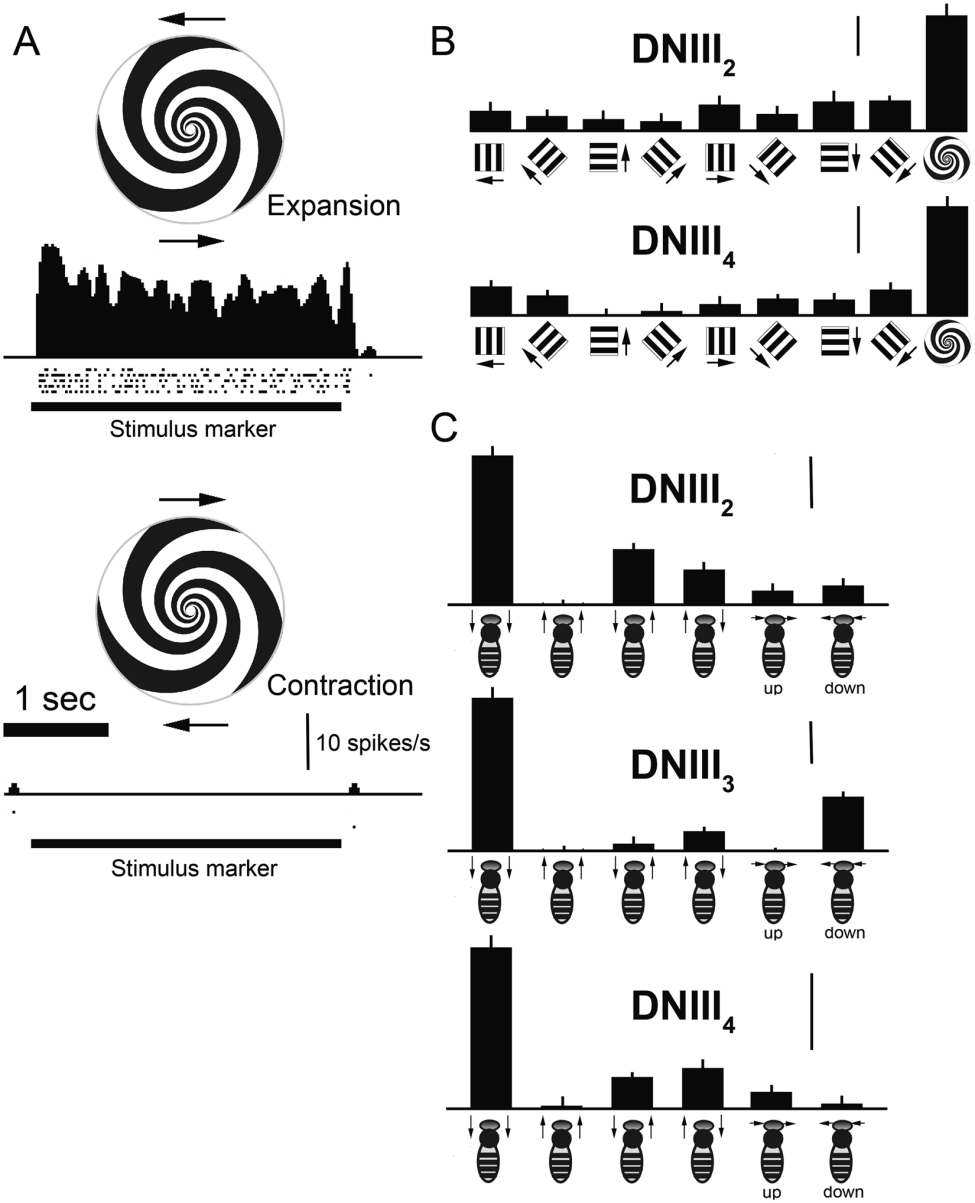
Directional tuning of DNIII neurons. (**A**) Response of a DNIII_4_ neuron to expansion and contraction of the spiral pattern. The stimulus was repeated 5 times: rasters show actual spike arrival times and the spike density function shows the general response waveform. The spiral rotated at 1.5 revolutions per second. (**B**) Responses of DNIII_2_ and DNIII_4_ neurons, confirmed through anatomical identification of the brain anatomies, to frontal stimulation with gratings and spirals. (**C**) Responses of anatomically confirmed DNIII_2_, DNIII_3_ and DNIII_4_ neurons to lateral stimulation, as indicated. Error bars are standard errors. The vertical bars in (**B**) and (**C**) show 10 spikes/s. The stimuli in (**B**) and (**C**) were standardized gratings of 0.05 cycles per degree moved at 20 Hz for 1 s. The depictions of the stimuli below the graphs are to guide the reader – they are not accurate depictions of the spatial frequencies used.

The DNIII neurons usually exhibit very low spontaneous activities (<1 spike/s). They spike vigorously when stimulated in their preferred motion direction and do not spike for anti-preferred motion (Fig. 4A). Due to the very low spontaneous rates, it is difficult to assess the level of anti-preferred inhibition. We have recorded responses to frontally located spiral patterns from anatomically confirmed DNIII_2_ and DNIII_4_ using 3 s periods of motion. Response amplitudes were measured as the mean spike rate in that 3 s motion period (which was followed by a 3 s rest period with no motion). We also recorded from several cells in which only partial fills were subsequently obtained, including examples that we strongly suspect as belonging to the DNIII_1_ and DNIII_3_ types. Unfortunately, their anatomies could not be definitively confirmed. Importantly, all of the DNIII cells that we have recorded from and identified as belonging to this general group consistently exhibit robust, ongoing responses to spiral rotation in a direction that generates visual expansion (upper, Fig. 4A). When the spirals are rotated in the opposite direction the neurons fail to spike (lower, Fig. 4A). For DNIII_2_ and DNIII_4_ we also stimulated frontally with gratings that moved in one coherent direction. While there was some variation in the responses to different directions of motion, the responses to spirals were significantly larger than responses to other types of frontal stimulation (Student t-test, p < 0.001) (Fig. 4B).

We recorded the responses to image motion presented on two laterally positioned stimulus monitors for cells confirmed anatomically as DNIII_2_ (n = 4), DNIII_3_ (n = 3) and DNIII_4_ (n = 3). We present the responses to simultaneous progressive image motion over both eyes, simultaneous regressive motion over both eyes, progression over the left and regression over the right eyes, and *vice versa*, and to simultaneous upward and downward motion over both eyes. It is evident that the neurons respond very strongly to simultaneous front-to-back motion over both eyes and do not respond to back-to-front motion (Fig. 4C). Responses to horizontal rotational motion also occur but these are relatively small (Fig. 4C). Only DNIII_3_ gave an appreciable response to downward image motion in the lateral regions of the eyes (Fig. 4C).

### Speed tuning with frontal spiral patterns

**Figure 5:**
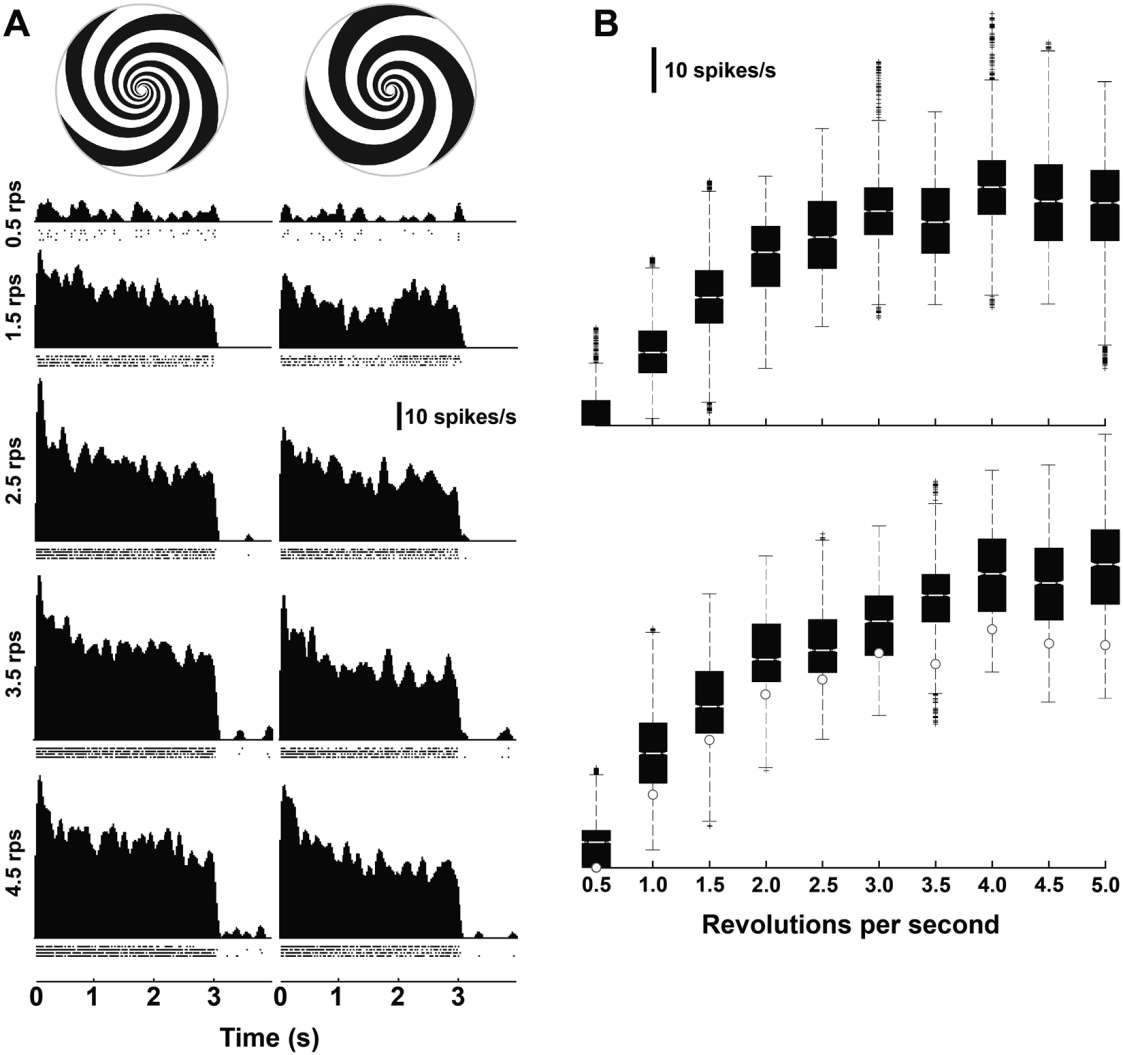
Speed tuning of DNIII neurons to frontal stimulation. (**A**) Spike density functions of a DNIII_2_ neuron to 2 different expanding spirals at different rotation speeds (4-arm spiral: left panel; 3-arm spiral: right panel). The stimulus ran from 0 to 3 s. (**B**) Box plots showing the median responses from recordings obtained from 8 DNIII neurons (including the cell in A) using two different spirals. On each box, the central mark is the median, the edges of the boxes are the 25th and 75th percentiles, the whiskers extend to the most extreme data points not considered outliers, and outliers are plotted individually (crosses). The upper panel is the 3-arm spiral and the lower the 4-arm spiral. The white dots in the lower plot show the medians for the 3-arm spiral. Abbreviations: rps: revolutions per second.

We present spike density functions obtained from an anatomically verified DNIII_2_ neuron stimulated with two expanding spiral patterns (3 and 4 spiral arms) rotated at five speeds (Fig. 5A). The spiral patterns were rotated at several rates ranging from 0.5 to 5.0 revolutions per second (rps), giving peripheral expansion speeds of 21.4°/s to 214.3°/s of expansion for both types of spiral arms. The spike density functions (see Methods) show that spike rates were maximal at stimulus onset and then decayed to a lower level over the first 1–2 seconds to a reasonably steady frequency. We recorded from a further 7 neurons that clearly belonged to the DNIII group but specific identities could not be determined due to missing dendrites or cell bodies in the fills. Therefore, we averaged the responses from all 8 cells together. The short recording time available using the intracellular technique allowed us to record 4–10 repetitions per stimulus condition per cell. Thus, for each revolution rate for each spiral pattern 32–80 data points were used to calculate the box and whisker plots shown in Fig. 5B. The median spike rate was calculated from the spike density function within the entire 3-second stimulus presentation time, which included the small onset transient and the relatively steady-state phase of the response. The median responses increased monotonically with pattern speed up to 3 revolutions per second for both spiral patterns and up to 4 revolutions per second for the four-arm pattern. The three-arm pattern responses are significantly lower than the responses generated by the four-arm pattern revolving at the same rate. This is indicated by the notches in the box plots, which do not overlap for a comparison between patterns at the same speed. We can thus conclude, with 95% confidence that the true medians differ significantly.

### Speed tuning with lateral stimuli

We also tested speed tuning during front-to-back lateral stimulation with gratings of various spatial (SF) and temporal frequencies (TFs). Due to the wide range of SFs and TFs used, we measured responses to 1 s of motion in the front-to-back direction. A 1 s rest period was allowed between presentations. With 9 TFs and usually 4 SFs, a single run took 72 s and 10 repeats were generally attempted (but not always achieved), leading to a 12 minute procedure. Onset transients were included in the response. As the TF was limited by the temporal properties of the screen, the range of TFs was always fixed. However, while we tried to maintain some consistency in SF, some cells clearly responded to very low SFs while others didn’t. Therefore the SF ranges we show vary slightly between cells, as selected by the operator during each recording session. Differences in the SF ranges used also restricted the type of statistical tests that could be conducted.

**Figure 6:**
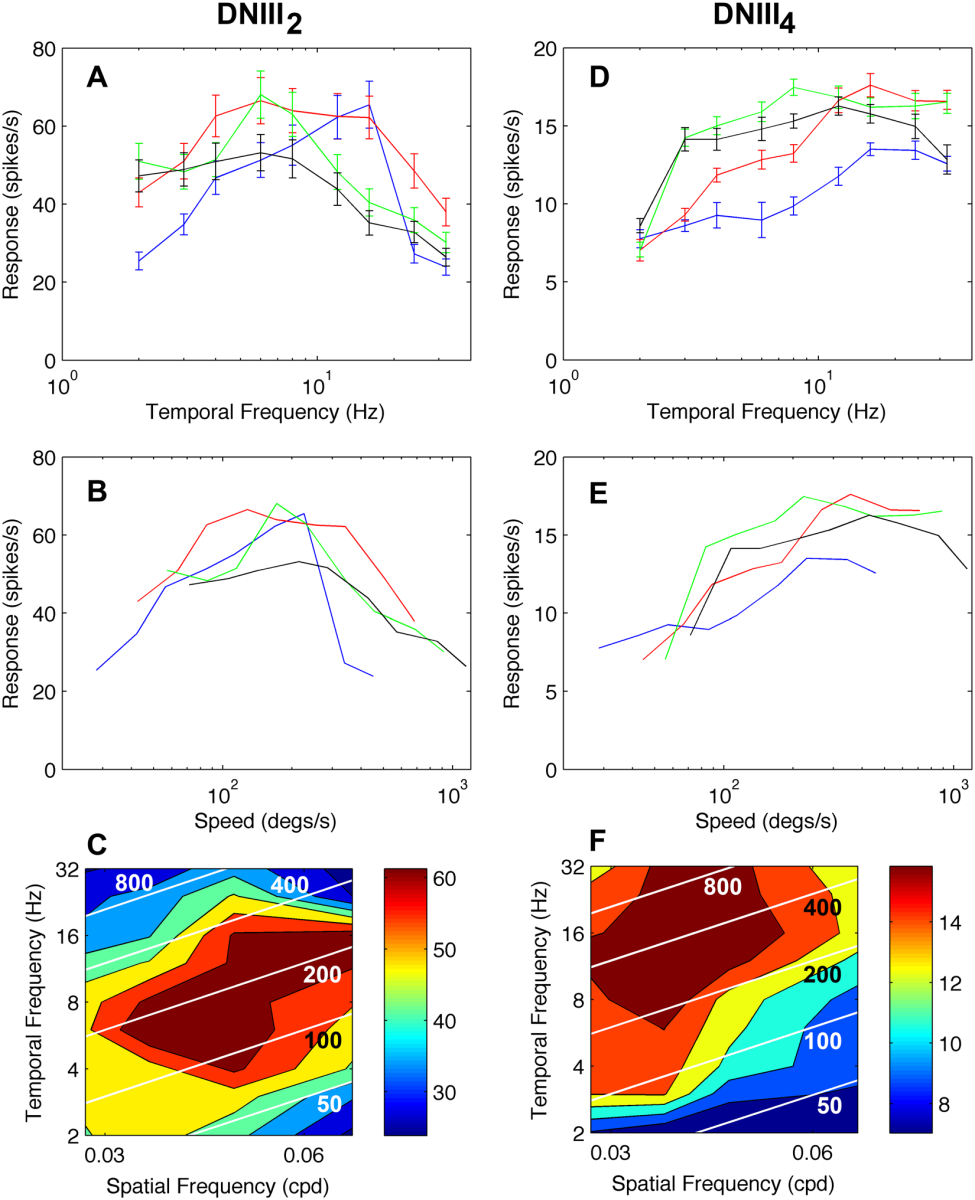
Speed tuning of two identified DNIII neurons. (**A**) Mean temporal frequency tuning for 3 DNIII_2_ neurons at four different spatial frequencies (black: 0.028 cycles/° (cpd), green: 0.035 cpd, red: 0.047cpd, blue: 0.071cpd). In this plot the mean values are the means across 3 high quality recordings. (**B**) The same data as in A but plotted with image speed on the x-axis. (**C**) Color-coded spatiotemporal contour map of the spike rate. The colors represent the spike rate as shown by the legend. The white lines represent constant image speeds (50, 100, 200, 400 and 800°/s). (**D**,**F**) The same format of figures for cell DNIII_4_. In this case the response presented was from one high quality recording during which all spatial and temporal frequencies were repeated at least 3 times. Error bars in A and D are standard errors.

DNIII_2_ was recorded from using the full spatiotemporal stimulus regime on 3 occasions (Fig. 6A–C). The mean peak tuning for the three cells did not align with a particular temporal frequency, as has been observed in other motion sensitive neurons in bees (Fig. 6A). For all spatial frequencies, the maximal response occurred in the speed range from 100–300 degs/s (Fig. 6B). Spatial frequency did influence the response amplitude but the speed tuning was remarkably resistant to large changes in spatial frequency. The contour plot (Fig. 6C) reveals that the optimum response zone occurs at spatial frequencies of 0.03–0.06 cpd and at temporal frequencies of 4–16 Hz. As we were able to record from these cells on multiple occasions we conducted a 2-way repeated measures analysis of spiking rate with SF and TF as the predicting factors. TF was the strongest significant predictor of spiking rate (F_(8,107)_ = 115,11, p < 0.0001). SF was also significant (F_(3,107)_ = 118.29, p < 0.0001), as was the interaction between SF and TF. The latter indicates that the effect of TF on spiking rate was different across SFs (F_(24,107)_ = 93.30, p < 0.0001). This is an indication that the speed of the stimulus, which is dependent on both the SF and TF of the grating was also important. To determine the relative strengths of SF, TF and their interaction, effect size was calculated using Eta^2^, which is the proportion of the total variability in spiking rate that is due to the variable of interest. We present this in percentage form in the following analysis. The Eta^2^ main effect of TF showed that 47.31% of the variability in spiking rate was due to TF alone. The Eta^2^ of the main effect of SF showed that 12.02% of the variability in spiking rate was due to SF alone. The interaction between SF and TF produced an Eta^2^ of 21.05%. Therefore, 80.38% of the variability in spiking rate was due to the SF and TF of the presented gratings. The remaining 19.62% of the variance was accounted for mainly due to differences between recording sessions (18.14%). The analysis for this cell type shows that SF, TF and their interaction (speed) are all significant predictors of the stimulus driven spiking rate.

**Figure 7:**
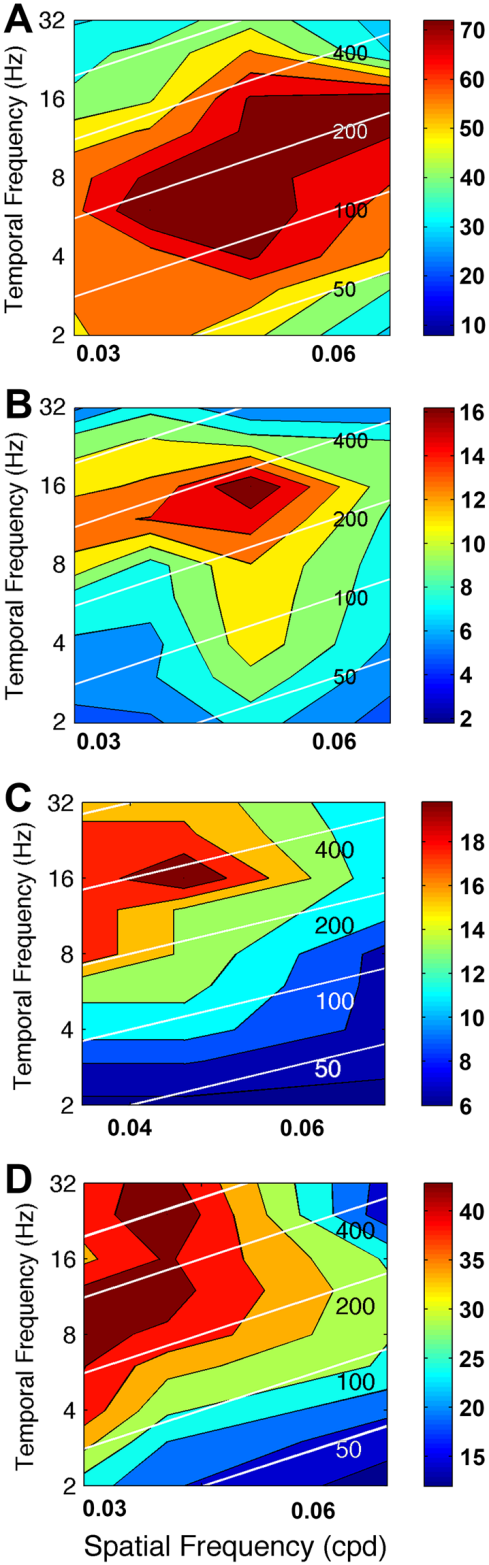
Spatiotemporal response plots for four DNIII neurons. (**A**) The spatiotemporal tuning of DNIII_2_ (as presented in Fig. 6C). This is repeated to allow direct comparison with the cells below. (**B**,**C**) Spatiotemporal response profiles for three neurons that were anatomically of the DNIII-type but without detailed dendrites, making specific identification difficult. The plots are arranged in such a way that the peak spatial frequency sensitivity declines from (**A**–**D)**. Therefore, the cell in (**D**) is tuned to lower spatial frequencies than the other three recordings. White diagonal lines indicate constant speeds, as indicated by the values printed on the plots (50, 100, 200, 400 and 800°/s). Color bars to the right indicate the mean number of spikes/s in the plots.

DNIII_4_ was recorded from using the side screens from three identified cells. However, a full spatiotemporal plot was only obtained from one cell, as presented (Fig. 6D–F). The cell responded strongly to a wide range of temporal frequencies. When the data was plotted against image speed it was evident that the cell was capable of responding at very high image speeds of 800–1000 degs/s (Fig. 6D–F). Spatial frequency influenced response amplitude but the cell gave robust responses at high speeds (>200°/s) at all tested spatial frequencies. The contour plot shows that the cell is maximally responsive at low spatial frequencies (0.03–0.05 cycles per degree) and high temporal frequencies (8–32 Hz). There was not sufficient data to conduct the same type of statistical analysis on this cell as we had done for DNIII_2_. Therefore, we combined the response of this identified cell with those of two complete spatiotemporal analyses obtained from other DNIII neurons where we could not clarify the cell identity (i.e. we combined the data in Fig. 6F and Fig. 7C,D). The rationale for combining these particular responses together is that all three recordings showed a sensitivity to low spatial frequencies and the same range of SFs was used. The statistical analysis was conducted as described for DNIII_2_ above. TF was the strongest significant predictor of spiking rate (F_(8,80)_ = 26.44, p = 0.0001). The main effect of SF was also significant (F_(2,80)_ = 26.44, p < 0.05). However, the interaction between SF and TF was not significant in these cells (F_(24,80)_ < 1, p > 0.05), probably indicating that speed was not a driving factor. The Eta^2^ for TF and SF showed that 16.36% and 14.78% of the variability in spiking rate was due to TF and SF, respectively. The interaction between SF and TF produced an Eta^2^ of only 2.52%. In total 33.66% of the variability in spiking rate was due to the SF and TF of the presented gratings. In these cells there is no evidence of any speed dependent modulation of spiking rate.

A high quality spatiotemporal tuning was obtained from another unidentified DNIII neuron (Fig. 7B). In this cell the main effect of TF was the strongest significant predictor of spiking rate (F_(8,143)_ = 4.08, p < 0.05) but the main effect of SF was not significant (F_(3,143)_ = 6.894, p > 0.05). The interaction between SF and TF was significant (F_(24,143)_ = 2.289, p < 0.01), suggesting that the speed of the stimulus was important. The Eta^2^ for TF and SF showed that 45.54% and 28.00% of the variability in spiking rate was due to TF and SF, respectively. The interaction between SF and TF produced an Eta^2^ of 10.03%. In total 83.57% of the variability in spiking rate after removing inter-subject variability was due to the SF and TF of the presented gratings. Thus, TF and the interaction between SF and TF (speed) were significant predictors of the stimulus driven spike rate.

### Response persistence

**Figure 8:**
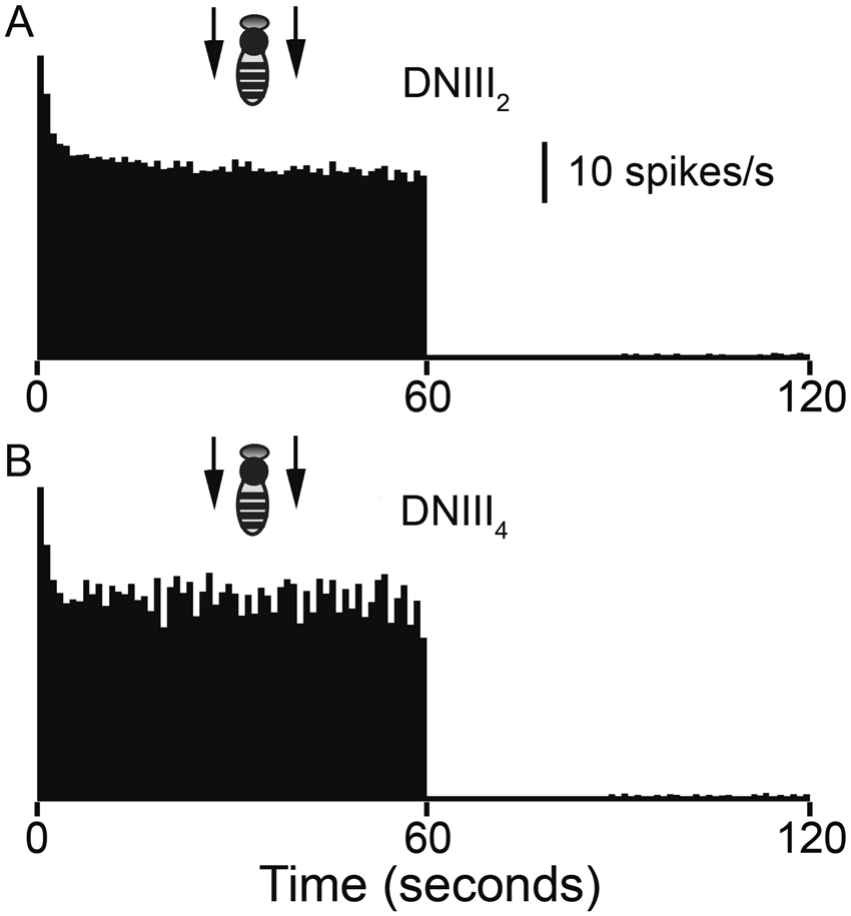
Response persistence. Peristimulus time histograms (PSTHs) showing the mean response amplitudes (6 repetitions in each cell) during 60 s of continuous grating motion at 200°/s for DNIII_2_ (**A**) and DNIII_4_ (**B**). Vertical bar = 10 spikes/s. The time bins in the PSTHs are 1 s in duration.

If the DNIII neurons are involved in monitoring flight speed during forward flights or during landing, the responses need to persist for the entire duration of the flight. Data in Fig. 5 has already shown that the DNIII neurons exhibit an initial onset transient followed by a steady state response for 3–5 seconds. We also examined the responses of several neurons to progressive motion presented for 60 seconds interleaved with 60 seconds of rest (in the latter, a gray stimulus of mean grating luminance was presented). This sequence was repeated six times for each cell. Figure 8 shows the responses of a DNIII_2_ and a DNIII_4_ neuron to 60 s of stimulation, followed by 60 s of recovery. The stimuli were laterally presented gratings that moved progressively at 200°/s (SF = 0.02cpd) over both eyes. The cells showed robust onset responses that decayed to a steady state level within the first 3 seconds. The response was then maintained at a steady level for the entire duration of the stimulus. After the stimulus was removed there was a period of at least 10 s where no spontaneous activity was observed. A low level of spontaneous activity was evident around 15 s after stimulus offset in both recorded cells.

## Discussion

### Cell classification

Goodman *et al*.^29^ provided drawings of the brain anatomies of three neurons in the bee that have similar structures to each other, which they labeled as DNIII cells. There is little doubt that the three cells they present belong to the same anatomical group of neurons that we describe here. All of the three neurons they present have dorsally positioned cell bodies, as is the case for DNIII_1_, DNIII_2_ and DNIII_3_. We introduce a fourth anatomically unique neuron in this general group that has its cell body located immediately posterior to its dendritic field in the lateral protocerebrum (DNIII_4_). From mass-fills we are certain that there are four neurons in the DNIII group because we never observed more than four axons in the bundle. Two other papers have mentioned DNIII neurons. One paper presents a drawing of the anatomy of a single DNIII neuron (which could be DNIII_1_, DNIII_2_ or DNIII_3_) in the brain and thoracic ganglia, but without depth information^31^. It also presents recordings that show that the neuron is maximally sensitive to front-to-back motion over the eyes, which correlates well with the data we present here. However, the speed range tested was restricted to 10–160°/s, which only covers the lower 10–20% of the speed range over which we have now shown these cells can respond. A second article shows that DNIII neurons respond strongly to frontal stimulation when the left half of the image moves leftward and the right half moves rightward, thus approximating the optic flow in the frontal field during forward motion^34^. Collectively, the previous works, combined with ours, show that the DNIIIs have consistent directional response characteristics. We have now expanded the knowledge of these neurons by presenting details of their anatomies, their ability to generate persistent responses to ongoing stimulation, and by revealing their speed tuning characteristics over a wide range of speeds both frontally and laterally.

### Morphology

The bee optomotor neurons have dendrites in the most posterior region of the brain, while those of the DNIII_4_ neuron (and another identified DNIII^31^) are more anterior and lateral^15^. While we have no information about the input from the optic lobes to the DNIII neurons in bees, it might be that their dendrites are positioned to maximize input from the anterior component of the lobula complex (often referred to as the ventral lobula using neuroaxis terminology) and its unique set of output neurons^39^. Strausfeld and Lee^40^ suggested two parallel DN pathways in flies: using neuroaxis terminology, DNs that innervate the ventral thoracic neuopiles receive input from the ventral lobula, while DNs supplying the flight motor receive input from the dorsal lobula. As at least some of the DNIII neurons send some of their axon terminals into the ventral thoracic neuropiles and may receive input from the ventral lobula, at first glance it appears that they could be part of a ventral system. However, their axons travel in the most dorsal tract in the ventral nerve cord (the medial dorsal tract^36^), which makes it difficult to class them as part of a ventral system. There are other direction selective DNs (e.g. DNIV) in bees that travel through more ventral tracts, such as the dorsal intermediate tract^30^.

In the thoracic ganglia, the terminals of the DNIIIs occupy neuropiles filled by the dendrites of both wing and leg motor neurons. The information on bee wing and leg motor neuron anatomy is not readily available in the published literature but is available in a doctoral thesis located at the University of London library^38^. In brief, the forewing nerves innervate the related muscles dorso-laterally through IIN1. Their somas and dendrites in the mesometathoracic ganglia (MMTG) give rise to many branches in both sides of the MMTG. Similarly, the hindwing nerve arises from neurons in the latero-dorsal region of the MMTG and extend dorso-laterally through IIN10 to enter the hindwing. Insects, including bees, need to carefully control the orientation of all body parts during flight, including the wings, head, legs and abdomen^5,41^. The optomotor system appears to stabilize the head and body during flight^41,42^, while front-to-back optic flow fields during periods of head stability control abdomen and leg angles to optimize streamlining^5^. During landing, bees also control their legs to ensure that the closest pair of legs touch down first^43^. It is, therefore, perhaps not surprising that neurons that are activated during visual expansion in the frontal visual field innervate both the dorsal wing and ventral leg motor neuropiles.

It is noteworthy that there are similarities in the general characteristics of the DNIII neurons in bees with those of the DNDC3–1 neuron in flies^18^. The DNDC3-1 neuron has anterior/lateral dendrites close to the exit of the lobula, a cell body close to the calyces and contralateral axons that descend into the thoracic ganglia. However, a point of difference is that DNDC3-1 has bilateral axon terminals, while the DNIII neurons have only contralateral axon terminals. The fly DNDC3-1 responds more strongly to frontal expansion than to frontal contraction, suggesting both physiological as well as anatomical similarities with the DNIII neurons in bees.

### Directional properties and response persistence

Bidwell and Goodman^34^ used a forward positioned, split screen stimulus to approximate the optic flow generated during forward and backward flight. The screen was divided into equal left and right halves, each containing a vertically oriented grating. They recorded from neurons that they classified as DNIII cells. They showed that image expansion (left in the left visual field and right in the right visual field) generated the maximal response in the cells, while contraction inhibited background activity. Clearly, this characteristic matches our findings using rotating spirals. We were able to extend these early observations by showing a range of expansion speeds, with patterns that had different spatial frequencies. We found that the cells increased their firing rates up to peripheral expansion speeds of around 150°/s, after which the response amplitude saturated. The cells were still responding robustly at peripheral expansion speeds of 214°/s. Using laterally positioned screens, we were also able to show that the cells were optimally responsive to front-to-back motion over both eyes, simulating the optic flow experienced during forward flight. Putting these findings together, it is apparent that the DNIII neurons carry signals appropriate for signaling forward body motion during forward flight or landing.

An essential characteristic of any neuron that has a role in controlling forward flight speed, steering or landing is that it must continue to signal image motion during long periods. We found that the DNIII neurons rapidly decreased their initial spike rate in the first 1–3 seconds after motion onset but then maintained very stable spike rates for the maximum duration that we tested (60 s). This response persistence appears to be optimal for a role in forward flight control. Srinivasan^12^ suggested that bees might integrate speed information during long flights to extract a signal that estimates how far they have flown – a so-called neural odometer. To do this, it is essential that they have an unerring speed signal throughout flight and the DNIII neurons appear well placed to provide such a signal.

### Speed tuning

Honeybees exhibit a number of flight behaviours that suggest an ability to calculate the angular velocity of images passing over their eyes, largely independently of the spatial frequency content of the environment^13^. Given this ability, the visual pathways that control flight speed must be able to measure image velocity over the eyes without the spatial texture of the environment having a large impact. Evidence in support of this notion is extensive, as outlined below. Honeybees use optic flow information to regulate flight speed: they tend to hold the speed of the image on the retina constant despite large changes in the textural information in the environment^3^. *Drosophila* have a similar ability to regulate their flight speed to hold a constant image speed over the eyes, regardless of large changes in image spatial frequency^44–46^. As already mentioned, the perception of distance flown depends on the amount of image motion experienced and this too is little affected by the contrast or the spatial texture of the image^47–51^. During centring responses, where bees are required to fly through narrow tunnels, they maintain equal distances between the walls by balancing the image velocities in the two eyes^52^. This centring response is also resistant to changes in image spatial frequencies. It has been suggested that a speed tuned system that is resistant to changes in the spatial structure of the image would be particularly useful in flying animals, where the spatial frequency content varies with the distance from objects^3^. For example, in open flight at relatively high altitudes forward flight speed would need to be high to maintain the expected image velocity, allowing bees to travel longer distances in a short time. Conversely, in dense visual environments the proximity of the surrounding structures would automatically adjust flight speed to lower, safer values. During landing on vertical or horizontal surfaces, the same realities hold: if a constant angular velocity is maintained over the eyes, the bee will slow down as it approaches the surface, improving safety and optimizing landing conditions. A gradual reduction in flight speed as bees approach landing sites is exactly what happens when bees land^8,7^.

The speed and directional tuning properties of several descending neurons in the bee brain have been described previously^15,30,31^. In all cases the neurons were optimally responsive for rotational image motion, i.e. as generated by body yaw, roll or pitch. Moreover, in all cases the cells were better described as being temporal frequency tuned than speed tuned. That is, the amplitudes of the responses were maximal at a particular temporal frequency for the majority of grating spatial frequencies tested: the optimum temporal frequencies were 8–10 Hz. In these ‘optomotor’ neurons, spatial frequency had a dramatic influence on response amplitude. The data from the DNIII neurons looks quite different. DNIII_2_ and DNIII_4_ respond broadly over a wide range of image speeds, the former continuing to respond strongly even at 100–300°/s and the latter having peaks of 800–1000°/s. These speed ranges are well within the normal image speeds experienced by bees during forward flight and landing. The spatial frequency of the stimulus did have a significant effect on the amplitude of most DNIII responses, so the system is clearly not a perfect speed tuned system, where speed sensitivity would be independent of spatial frequency. It was the case that the interaction between temporal and spatial frequency was also significant for some cells, suggesting that speed was a controlling factor on spike rate. The speed tuning functions of the cells are broad enough across a range of spatial frequencies that the responses are quite resistant to large changes in spatial structure. Ibbotson^53^ reported some extracellular speed tuning functions from bee ventral nerve cord that qualitatively resemble those of the DNIII_4_ neuron, suggesting a link between the extracellular recordings and DNIII_4_.

Bees approaching to land on a vertical surface maintain a constant rate of expansion of about 220°/s at a viewing angle 45° from ‘straight ahead’, which is the direction in which the rate of expansion is maximal^6^ (also see Fig. S1). It is useful to examine how this stimulus relates to the responses exhibited by the DNIII neurons in our experiments. Using equation (S2) in the supplementary information, we estimate that the stimulus experienced by the landing bees corresponds to an expansion rate of about 175°/s at the periphery of our spiral stimulus for the highest rotational speed (5 rps). Inspection of the responses in Fig. 5 shows that this is the domain in which the median DNIII neuron responses exhibit the highest spike rates. Our experiments with the translating linear gratings indicate that the DNIII_4_ responses exhibit a broad maximum at image velocities in the range of 200–600 °/s, which is again close to the velocities that bees use to regulate their cruising speed and streamline their abdomen^3,5,6,8^.

Thus, it is evident that the DNIII neurons exhibit strong responses at image velocities that are relevant in the context of cruising flight as well as landing, which makes these neurons attractive candidates for the sensory control of these behaviors. However, the neural control of flight speed during cruise or landing requires not only strong responses to the relevant patterns of optic flow, but also a good sensitivity to changes in the speed of image motion. This is because it is the change in speed that needs to be sensed and corrected. Our data suggest that the DNIII neurons will be working in a range in which the responses are strong, but not very sensitive to small changes in image speed. However, there is now increasing evidence that motion-detecting neurons in the insect visual pathway are much more sensitive during the execution of real behaviors, as opposed to situations in which the insect is immobilized for recording purposes. Recordings of movement-detecting neurons from tethered flying or walking insects (or from brains with heightened levels of octopamine, a neuromodulator which mimics the ambience of natural flight) reveal a shift of the speed tuning curves toward higher image speeds^54–58^. It is conceivable, therefore, that during real flights that elicit image speeds of 200–400°/s, the honeybee’s DNIII neurons are operating in a region where the tuning curve displays a steep slope, and confers a high sensitivity to changes of image speed. It is also possible that the sensing of image speed is performed not just by the DNIII neurons but by a population of motion-sensing neurons with a variety of speed tuning curves, in which case the image speed could be estimated precisely by comparing responses across the neural population.

## Electronic supplementary material

Supplementary information
