# Supplementary material for: Neural basis of forward flight control and landing in honeybees
Source: Sci Rep. 2017 Nov 6;7:14591. doi: 10.1038/s41598-017-14954-0 (PMC5673959; doi:10.1038/s41598-017-14954-0)
Supplement: Supplementary file 1 — Supplementary information [file 41598_2017_14954_MOESM1_ESM.pdf]

Supplementary Information

Neural basis of forward flight control and landing in honeybees

Ibbotson MR, Hung Y-S, Meffin H, Böddeker N, Srinivasan MV

Supplementary Information

Spiral stimuli

The spirals used were exponential spirals, whose contours are described by the polar function

$$R_i(\psi) = Ae^{B(\psi-\psi_i)}\,, \quad \psi > \psi_i \qquad (S1)$$

Here $R_i(\psi)$ specifies the radius of the *i*th contour (i=1,2…n), as a function of angle ψ. The total number of contours (n) in the spiral pattern is twice the number of arms (black or white stripes). A is the initial radius of the spiral contour, B is the (exponential) pitch of the spiral, and $\psi_i = \frac{2\pi i}{n}$ specifies the commencing angle of contour *i*. The reason for the use of an exponential spiral (as opposed to an Archimedian spiral, for example) is that a rotating exponential spiral generates exactly the same pattern of optic flow that a visual system would experience when it approaches a stationary vertical surface (Baird et al, 2013). Spirals with 3 or 4 arms (n= 6 or 8) were used (Fig. 1A), rotating at 1-5 rps, with a pitch of 0.3.

It can be shown that the angular velocity of expansion α (rad/s) of the image of a spiral with exponential pitch B, rotating at *f* revolutions per second, along a viewing direction θ is given by (Baird et al, 2013):

$$\alpha = B\pi f \sin 2\theta \qquad (S2)$$

where $\theta = 0$ represents the 'straight ahead' direction. A plot describing the variation of α with the viewing direction θ is shown in Fig. S1. We note that this expansion profile depends on the pitch (B) and the rotational speed (*f*) of the spiral pattern, but is independent of the distance to the spiral pattern or the number of arms that it carries.

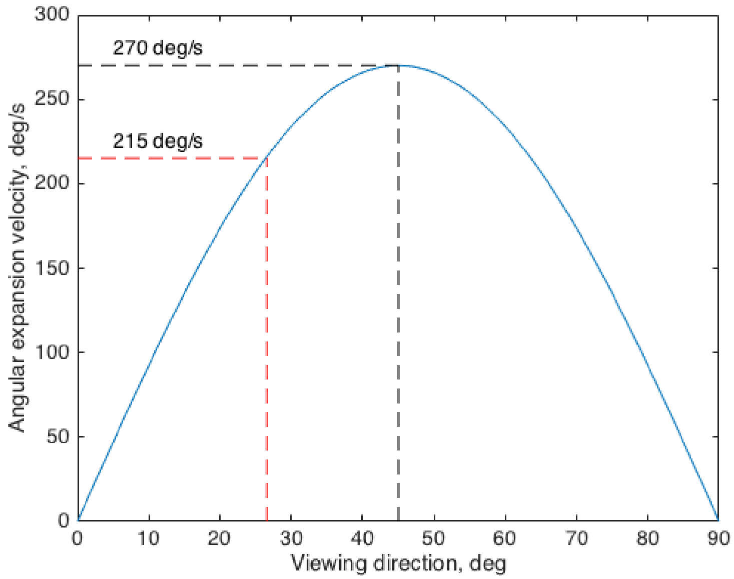

Figure S1. Variation of the rate of angular expansion with viewing direction of the image created by an exponential spiral, with pitch B=0.3, rotating at 5 rps, calculated using equation S2. A viewing direction of 0 deg corresponds to the 'straight ahead' direction. The maximum rate of expansion (270 deg/s) occurs in the viewing direction of 45 deg, as shown by the black dashed lines. The rate of expansion at the outer boundary of the spiral stimulus used in our experiments (corresponding to a viewing direction of 27 deg) is 215 deg/s.
